# Initial solvent conditions impact the reproducibility and aggregation trajectory of monomeric Amyloid Aβ

**DOI:** 10.1002/alz.089897

**Published:** 2025-01-03

**Authors:** Zahra Najarzadeh, Charles G Glabe

**Affiliations:** ^1^ University of California, Irvine, CA USA; ^2^ University of California Irvine, Irvine, CA USA

## Abstract

**Background:**

Our goal is to identify conditions to produce structurally homogeneous and reproducible preparations of different polymorphic structures. Here we investigate the effect of several widely used methods for solubilizing Abeta on the subsequent aggregation process.

**Method:**

Aliquots of HPLC‐purified synthetic Aβ40 in originally lyophilized from acetonitrile/water (AcN) 50% v/v were dissolved in hexafluoroisopropanol (HFIP) 100%, AcN 50% v/v, NH4OH 2%, or 50 mM Phosphate buffer (PB), re‐aliquoted and lyophilized. These aliquots were dissolved in 2% NH4OH, HFIP, AcN 50%, DMSO or 50 mM NaOH to make a stock solution (4mM) or directly dissolved in PB to a final concentration of 50µM. Fibrillation kinetics assays were conducted in triplicate after dilution of the stock solutions 50 uM in PB and fibrillization monitored by thioflavin‐T (ThT).

**Result:**

We observed large differences in the aggregation behavior of Aβ depending on initial solvent conditions, impacting lag phase, ThT kinetics, fluorescence end point, fibrilization rate, and overall reproducibility. Solubilization in 50 mM NaOH significantly enhances the reproducibility of fibrillation compared to other methods confirming previously published work. Initial solubilization HFIP and NH4OH for stock preparation yields less reproducible aggregation behavior. Dissolving peptide in NH4OH leads to a longer lag phase (almost 10 h longer) compared to using other solvents. Directly dissolving the peptide in PB demonstrates better reproducibility compared to preparing stock in solvents like HFIP, NH4OH, AcN50%, and DMSO. Additionally, we demonstrate that sonication and centrifugation of the stock before dilution contribute to achieving an aggregate‐free monomeric solution. Even after monomerization in 50 mM NaOH, prior treatment with other solvents results in reproducible differences in aggregation behavior, suggesting that the monomeric peptide may retain local structure imprinted by prior solvent exposure.

**Conclusion:**

Our analysis indicates that solubilization with NaOH (50mM) offers a dependable method for solubilizing Aβ monomer. This results in monomeric and homogeneous peptide stock solutions, exhibiting exceptionally reproducible assembly kinetics when returned to near‐neutral pH. Prior solubilization and monomerization of Aβ in HFIP, DMSO, AcN and NH4OH results in variable kinetics and extent of fibrillation. Prior exposure to these other solvents produces reproducible differences in aggregation even after monomerization in NaOH.

